# Operation for Hemorrhoids

**Published:** 1907-08

**Authors:** William S. Goldsmith

**Affiliations:** Atlanta, Ga.


### OPERATION FOR HEMORRHOIDS.*

BY WILLIAM S. GOLDSMITH, M. D., ATLANTA, GA.

Diseased conditions about the rectum are regarded by the public with much prejudice and superstition.

The operation for hemorrhoids has long been condemned by the unthinking public, as a procedure which carries with it a possibility of shortening the life of the individual. Long standing fistulae have been allowed to exist through the fear of complications which do not and cannot arise. The removal of hemorrhoids by the method of ligation or by use of the clamp and cautery, are the means to an end which I think are rapidly being relegated to obscurity. To one who has observed the suffering and slow convalescence, and not infrequently ulcers, which follow the operation of ligation, could not but be impressed with any operation which seeks to attain perfect relief with a minimum of pain, and certainly with an immensely shorter period of convalescence. To an extent, though not so marked, the same objections obtained with the clamp and cautery operation.

For the past three years I have been operating exclusively by the operating of excision. I claim no credit for originality for the principle of the operation, and the only matter of interest is some small points of technique. The patent is prepared in the usual way, preferably by being purged by castor oil, followed by an enema. After complete anesthesia has been secured and through divulsion of the spincter is accomplished with the thumb or fingers of the operator, and not, as is sometimes done, with an instrument. The rectum is

*Read before the Fulton County Medical Society.

again irrigated with a saline solution, after which a gauze tampon with a string attached, is introduced three or four inches into the rectum which acts as a very serviceable plug in keeping the field of operation dry and clean. The hemorrhoidal tumor is caught with a pair of forceps, delivered, and the base on either extremity of the flattened tumor is also grasped with a pair of hemostatic forceps. The hemorrhoid is then dissected clean and the spurting, bleeding points are clamped with forceps and ligated with fine plain catgut. I should say in this connection that the dissection of the tumor is in the long axis of the gut and the lines of other excisions are directed along the same axis; this point, of course, is perfectly obvious as, if the dissections were carried transversely or following the circumference of the gut, danger of a certain amount of stenosis would occur. After the removal of the hemorrhoid by this method, the exposed wound area is closed by the introduction of a No. 00 chromic catgut. I have found that the matter of the introduction of this suture is of some importance, in that the small bite of tissure in the floor of the wound area is incorporated with the suture which serves to destroy the possibility of the occurrence of a dead space. The interrupted suture also permits the free drainage through the incision line between the sutures, thereby preventing the oedema often seen about rectal wounds. I find that it is the experience of all of us who have operated for hemorrhoids by whatever method, to have as a result, a number of mucous tabs, which leads the patient to feel that something has been left undone during the operation. This condition can be overcome by extending the hemorrhoidal incision in the direction of the muco-cutaneous junction and closing this wound area with an interrupted chronic catgut. Theserectal wounds require practically no dressing and but for a gauze pad I use nothing. After the recovery from the probable nausea of the anesthetic, I put these patients on a full diet, having the bowels to move after forty-eight hours after the operation, and every second night thereafter, administering a mild laxative pill sometimes followed by an oil enema, using a small rubber catheter to facilitate the introduction of the oil. This small chromic catgut can safely be relied upon to remain in position for ten days or two weeks, and the patient can be assured that the sutures will be thrown off by the process of absorption. The objection to the continuous suture, either plain or chromic catgut, is that it tends to act as a ligature and much oedema will follow and the probability of strangulation necrosis is increased.

